# Review—Emerging Portable Technologies for Gait Analysis in Neurological Disorders

**DOI:** 10.3389/fnhum.2022.768575

**Published:** 2022-02-03

**Authors:** Christina Salchow-Hömmen, Matej Skrobot, Magdalena C. E. Jochner, Thomas Schauer, Andrea A. Kühn, Nikolaus Wenger

**Affiliations:** ^1^Department of Neurology With Experimental Neurology, Charité–Universitätsmedizin Berlin, Berlin, Germany; ^2^Control Systems Group, Technische Universität Berlin, Berlin, Germany; ^3^Berlin School of Mind and Brain, Charité–Universitätsmedizin Berlin, Berlin, Germany; ^4^NeuroCure Clinical Research Centre, Charité–Universitätsmedizin Berlin, Berlin, Germany; ^5^German Center for Neurodegenerative Diseases, DZNE, Berlin, Germany

**Keywords:** motion tracking, human kinematics, locomotion, postural control, wearables, digital image processing, Parkinson's disease, multiple sclerosis

## Abstract

The understanding of locomotion in neurological disorders requires technologies for quantitative gait analysis. Numerous modalities are available today to objectively capture spatiotemporal gait and postural control features. Nevertheless, many obstacles prevent the application of these technologies to their full potential in neurological research and especially clinical practice. These include the required expert knowledge, time for data collection, and missing standards for data analysis and reporting. Here, we provide a technological review of wearable and vision-based portable motion analysis tools that emerged in the last decade with recent applications in neurological disorders such as Parkinson's disease and Multiple Sclerosis. The goal is to enable the reader to understand the available technologies with their individual strengths and limitations in order to make an informed decision for own investigations and clinical applications. We foresee that ongoing developments toward user-friendly automated devices will allow for closed-loop applications, long-term monitoring, and telemedical consulting in real-life environments.

## 1. Introduction

The widespread application of technologies for gait analysis has contributed greatly to our current understanding of healthy and pathological locomotion (Celik et al., 2021). On one hand, instrumented gait analysis complements the quantification of long-established clinical scales [e.g., Berg Balance Scale (Berg et al., 1989), Timed-up-and-go test (Podsiadlo and Richardson, 1991)] and patient self-reports [e.g., Freezing of Gait Questionnaire (Giladi et al., 2000)]. On the other hand, portable technologies for gait analysis may improve diagnosis, follow-up, and treatment of gait disorders through continuous monitoring in activities of daily living (Tzallas et al., 2014; Filli et al., 2018; Ancona et al., 2021). In concert with functional neuroimaging and neuromodulation, gait analysis technologies can enhance our knowledge of healthy and pathological gait function (Maetzler et al., 2009; Artusi et al., 2018; Buckley et al., 2019).

Gait and postural control disorders in the context of neurological diseases, such as Parkinson's Disease (PD) and Multiple Sclerosis (MS), have an immense impact on affected people's quality of life (Snijders et al., 2007). Parkinson's Disease is the second most common neurodegenerative disease in the elderly in Europe (Deuschl et al., 2020). Patients frequently suffer from slow movements (bradykinesia), pathological gait patterns including reduced step length and freezing of gait (FoG) (Nutt et al., 2011), as well as difficulties in postural control (Schlenstedt et al., 2016). Numerous publications have shown that gait parameters extracted with optical motion capturing, force plates, or inertial sensors correlate with clinical assessments of disease severity and levodopa responsiveness in PD (Horak and Mancini, 2013). In patients with MS, leg paresis, loss of coordination, and spasticity often manifest as gait dysfunction and changes in balance control (Comber et al., 2017). MS related gait and balance impairments can occur at very early disease stages, however, so minor that they may be difficult to see with the bare eye (Kieseier and Pozzilli, 2012). Advanced movement analysis techniques can measure these subtle changes and could be used to identify the risk of mobility loss (Shanahan et al., 2018). In this narrative review on gait analysis technologies in neurological disorders, we will discuss PD and MS as illustrative examples because of their distinct gait characteristics and clinical relevance in different age groups.

Recently, there has been a growing research interest in contact-free human motion tracking with available clinical equipment (e.g., standard camera) using machine learning (ML). Telemedical patient care options play an increasing role in times of global pandemics (Sibley et al., 2021). Therefore, the literature published on the topic of reliable, easily accessible, and easy to use measurement systems for gait and balance analysis is extensive and can be overwhelming. This narrative review aims at building a technical understanding of emerging portable gait analysis technologies for neurological disorders, which can be classified into non-wearable and wearable devices (*wearables*) (de-la Herran et al., 2014). Our goal is to enable the reader to understand the strengths and limitations of available technologies and thereby support decision-making for planning applications in research and diagnostics. After briefly introducing gait and postural control measures, wearable and recent non-wearable systems from the last decade are discussed in detail for their functionality, usefulness, and usability in practice. Future applications and trends are identified.

## 2. Measures of Gait and Postural Control

### 2.1. Gait Measures

**Figure 1:**
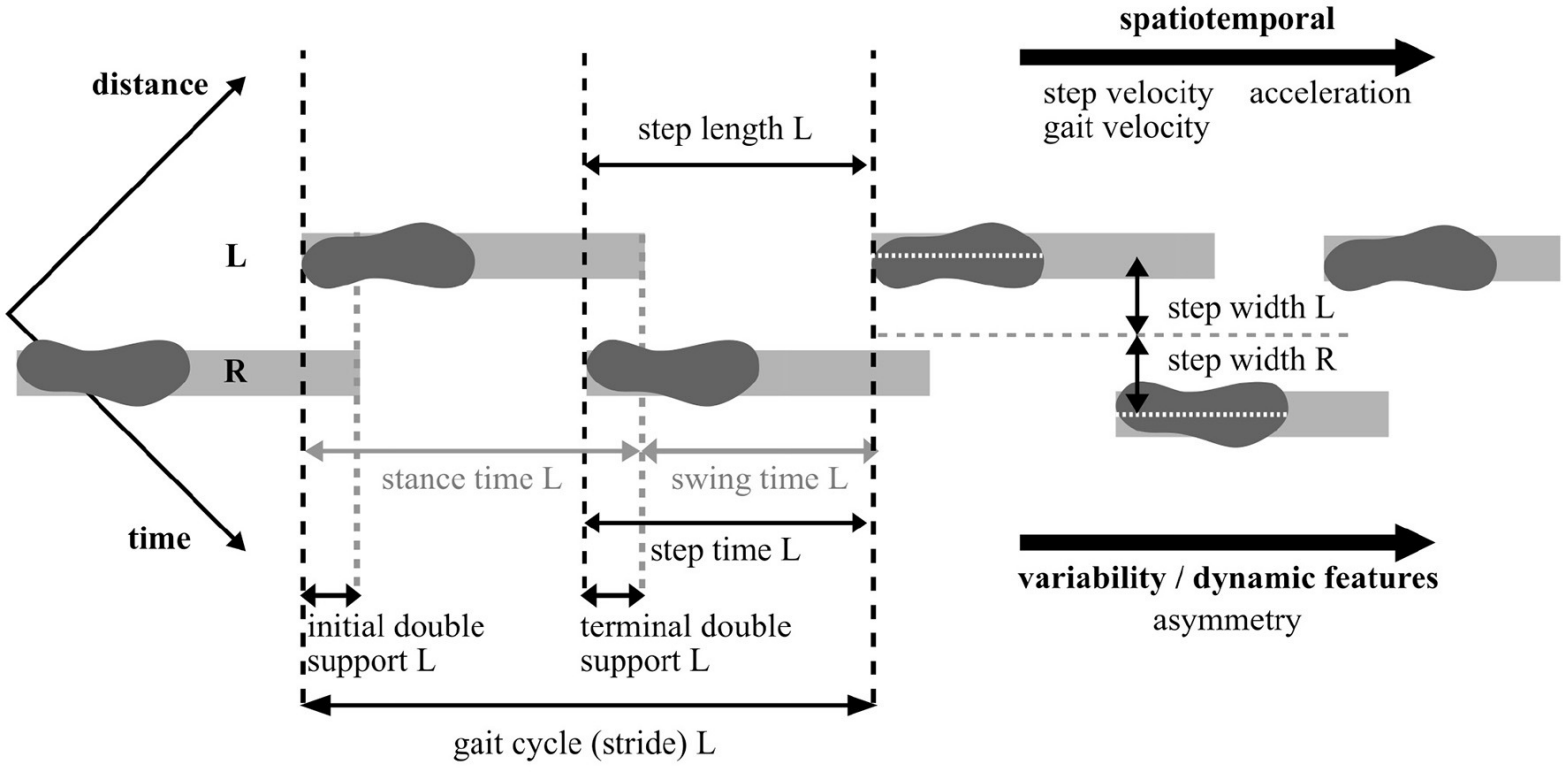
Illustration of basic spatiotemporal and dynamic gait parameter definitions. Note that the footprint indicates the heel strike event. L, Left foot; R, Right foot.

Gait results from cyclical limb movement. For its analysis, parameters are often defined in the dimensions time and space, as illustrated in Figure 1. Despite the two displayed main phases, stance phase and swing phase, the gait cycle can be divided into up to eight phases with regard to leg position, foot position, and load (namely initial contact, loading response, mid stance, terminal stance, pre-swing, initial swing, mid swing, and terminal swing) (Taborri et al., 2016). Resulting spatiotemporal features such as gait cycle and gait velocity are commonly expressed as the average of several strides. Dynamic features of gait represent the stride-to-stride variability of these measures in the form of intra-subject standard deviation or coefficient of variation (Lord et al., 2013; Buckley et al., 2019). Further variables can be extracted such as parameters in the frequency domain or sub-task-specific parameters, e.g., the rotational velocity of turns (Horak and Mancini, 2013). The evaluation of sub-tasks in standard clinical tests may be relevant for investigating specific symptoms. For example, sequential tasks of turning and passing through narrow doors have been designed to provoke episodic FoG symptom in PD (Ziegler et al., 2010; Reches et al., 2020). In addition, kinematic parameters such as knee joint angles or range of motion (ROM) as well as kinetic parameters such as ground reaction forces (GRF) can be extracted from certain portable systems to monitor disease progression (Baker, 2013; Veeraragavan et al., 2020). A list of frequently reported gait and balance parameters is presented in Table 1.

**Table 1:** Examples of commonly derived measures of gait and postural control from instrumented analysis technologies.

**Parameter**	**Unit**	**Examples PD**	**Examples MS**
**Spatiotemporal, kinematic gait parameters**			
Gait cycle / stride duration	s, ms	Blin et al., 1990; Ginis et al., 2017; Shah et al., 2020	Benedetti et al., 1999; Straudi et al., 2013; Müller et al., 2021
Cadence	steps/min	Curtze et al., 2015; Horak et al., 2016; Iijima et al., 2017	Martin et al., 2006; Straudi et al., 2013; Leone et al., 2018
Gait velocity / speed	m/s, cm/s	Herman et al., 2014; Galna et al., 2015; Fino and Mancini, 2020	Benedetti et al., 1999; Remelius et al., 2012; Müller et al., 2021
Stride / step length	m	Rochester et al., 2014; Ferrari et al., 2016; Cebi et al., 2020	Martin et al., 2006; Remelius et al., 2012; Leone et al., 2018
Double support time	% cycle, % stride	Blin et al., 1990; Curtze et al., 2015; Shah et al., 2020	Benedetti et al., 1999; Straudi et al., 2013; Leone et al., 2018
Stride / step time variability	s	Herman et al., 2014; Galna et al., 2015; Ma et al., 2020a	Moon et al., 2015; Allali et al., 2016; Kalron et al., 2018
Knee (lower leg) ROM	degree	Dewey et al., 2014; Curtze et al., 2015; Horak et al., 2016	Rodgers et al., 1999; Filli et al., 2018; Valet et al., 2021
**Postural stability parameters**			
Postural sway area / range	m/s, cm	Mancini et al., 2012a; Dewey et al., 2014; Horak et al., 2016	Spain et al., 2012; McLoughlin et al., 2014; Solomon et al., 2015
Postural sway jerk	m^2^/s^5^	Mancini et al., 2012a; Dewey et al., 2014; Horak et al., 2016	Sun et al., 2018; Arpan et al., 2020; Gera et al., 2020
RMS amplitude	m/s, cm	Mancini et al., 2012b; Nantel et al., 2012; Chen et al., 2018	Sun et al., 2018; Santinelli et al., 2019; Arpan et al., 2020

*RMS, Root mean square*.

*Three exemplary publications are listed for each parameter. Note that the exact parameter definition might vary between references*.

With the high amount of redundant and covariant available parameters from instrumented gait analysis, methods have been suggested to summarize parameters for better interpretation: For example, the *Gait Variability Index* (GVI) was introduced by Gouelle et al. (2013) combining nine weighted gait parameters based on results of a principal component analysis (PCA) in comparison with a reference group. Morris et al. (2017) proposed a model of unrestricted gait based on data from wearables on 103 elderly controls and 67 PD patients. Four gait domains were derived from 14 gait parameters by applying a PCA: pace, rhythm, variability, and asymmetry. Further models are summarized in Celik et al. (2021). Although simplifying the complexities of instrumented gait assessment would be helpful, the prevalence of these higher-order parameters in clinical trials has been low to date, possibly due to the complexity of their analysis and interpretation.

Since the number of kinematic gait analysis technologies has grown excessively in recent years, we focus on the assessment of kinematic parameters in this review. However, additional investigations of the phasic contribution of muscles in a gait cycle can be obtained from surface electromyography (EMG), integrated into many studies on human locomotion and neurological disease characteristics (e.g. Winter, 1989; Mickelborough et al., 2004; Gnther et al., 2019; Cofré Lizama et al., 2020). A detailed overview of standardized clinical tasks and protocols for the assessment of gait, such as the timed 10m walking test or the timed-up-and-go test (TUG), can be found in Graham et al. (2008) or de-la Herran et al. (2014).

### 2.2. Balance Measures

**Figure 2:**
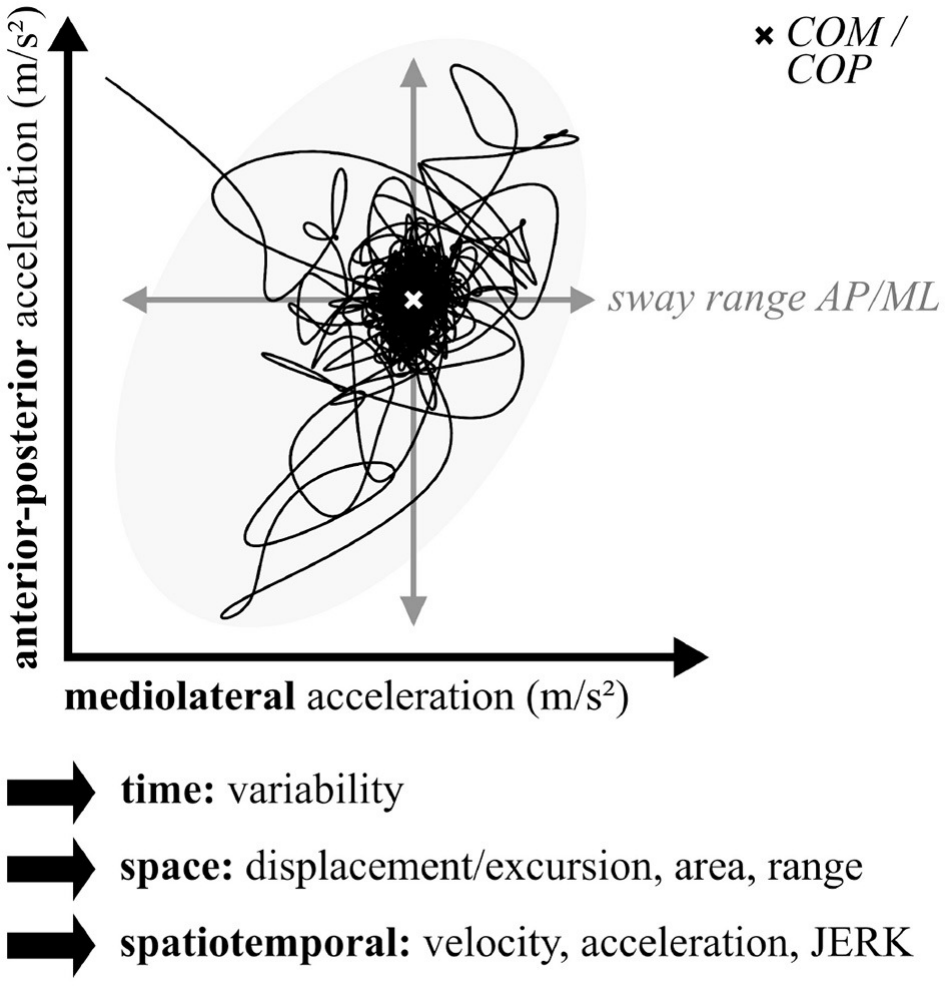
Balance and postural sway parameters.

Depending on the measurement modality, either the center of pressure (COP) or the center of mass (COM) is tracked in balance assessments during standing in different conditions (Buckley et al., 2019). Often utilized conditions are standing on hard surfaces vs. foam surfaces [modified clinical test of sensory interaction on balance (Horn et al., 2015)] or eyes-open vs. eyes-closed. Multiple parameters are determined describing the displacement of COP or COM over a defined amount of time as illustrated in Figure 2. Postural sway captures the horizontal acceleration of the person's center in all directions, most often in the mediolateral and anterior-posterior planes. Typically, sway area, sway range, sway velocity, and jerk, defined as the smoothness of the trunk sway (rate of change), are extracted and analyzed regarding asymmetry and variability between different conditions (Martinez-Mendez et al., 2012).

Balance and gait may represent independent domains of mobility in neurological diseases (Horak et al., 2016). Thus, no single measure of either balance or gait can fully characterize mobility impairments, although gait parameters facilitate statements on the balance capabilities of a person. Longer stance phases, expanded step width and deviations from walking a straight line were reported in people with balance disorders (Spain et al., 2014; Diaz et al., 2020).

### 2.3. Measures/Biomarkers in PD

Parkinsonian gait differs from the gait of the healthy elderly even in the early stages of the disease as revealed by kinematic measures. Galna et al. (2015) found an impairment across the gait domains pace, variability, rhythm, asymmetry, and postural control in recently diagnosed PD patients compared to age-matched healthy controls. PD patients walked at a slower pace, with decreased step length, and showed increased asymmetry and step-to-step variability. Others reported a set of 20 gait kinematic variables, such as stride length or gait velocity, that differentiates parkinsonian gait from the gait of controls, and a set of variables correlating with symptom severity, potentially serving as markers of PD progression (Dewey et al., 2014). Recently, Ghislieri et al. (2021) used foot-switch sensors to assess gait parameters in PD patients and age-matched controls during walking and reported a 42%-increase in atypical gait cycles in PD, which correlated with motor symptom severity^1^. Veeraragavan et al. (2020) showed that a neural network approach with features extracted from the vertical ground reaction force can differentiate PD from controls as well as predict disease severity (Hoehn & Yahr stage). Furthermore, postural instability is increased in early PD and deteriorates within 12 months of diagnosis, thus providing a potential marker for motor function decline (Mancini et al., 2011, 2012a). Increased gait variability and sagittal trunk movement might predict an increased risk of falls (Ma et al., 2020a). Yet, no standardized set of gait kinematic biomarkers that signifies gait improvement in PD exists (Horak and Mancini, 2013).

### 2.4. Measures/Biomarkers in MS

Differences in gait and balance parameters between neurologically intact controls and MS patients were reported (Shanahan et al., 2018): Reduced gait speed and stride length, a prolonged double support time, as well as changes in kinematic characteristics of the hip, knee, and ankle joint were found to correlate with disease severity in patients with relapsing-remitting, primary or secondary progressive MS with no to moderate impairments^2^ (Benedetti et al., 1999; Martin et al., 2006; Kelleher et al., 2010; Remelius et al., 2012). Additionally, a relationship between a reduced dorsiflexion angle at initial contact and walking induced fatigue as well as an increased power absorption at the hip, knee, and ankle have been reported in MS patients with moderate disabilities (EDSS 3–6) (McLoughlin et al., 2016). Studies on balance in MS patients with mild to moderate impairments (EDSS 0–5.5) showed an increased mediolateral sway path length, mediolateral sway range (Solomon et al., 2015), and sway area (Spain et al., 2014). This is also reflected by a wider stride width in patients with EDSS 2.5–6 compared to controls (Remelius et al., 2012). Clinical tests including turns were recommended to reveal important markers of balance confidence and walking abilities in MS (Adusumilli et al., 2018).

## 3. Wearable Technologies

### 3.1. Inertial Sensors

#### 3.1.1. Technology

The progress in micro-electromechanical system (MEMS) technology resulted in the availability of small, lightweight, and low-cost inertial measurement units (IMUs) conquering the motion tracking market (Seel et al., 2020). Therefore, IMUs are the most widely used type of wearable sensors for gait and balance analysis (de-la Herran et al., 2014; Shanahan et al., 2018); the quantification of gait with IMUs is sometimes referred to as InertiaLocoGraphy (ILG) (Vienne-Jumeau et al., 2020). IMUs typically consist of a combination of multi-dimensional gyroscopes, accelerometers, and often magnetometer sensors allowing the estimation of joint angles, gait and angular velocities, position and orientation in space via sensor fusion techniques (Sabatini, 2006). The wireless sensors can be mounted on various parts of the patient's body, for example, foot, lower leg, pelvis, torso, or integrated into garments and insoles, in order to measure movements of a specific body segment.

*Accelerometers* are most commonly used in motion analysis and assess the one-, two-, or three-dimensional acceleration of the sensor in terms of externally applied acceleration forces (Diaz et al., 2020). The measured signal is the sum of (1) the linear acceleration, namely the translation- and/or rotation-related instantaneous change of velocity, and (2) the earth's gravitational acceleration, which is approximately 9.81 m/s^2^ in vertical direction near the earth's surface. However, these two components can only be differentiated completely in quasi-stationary scenarios.

*Gyroscopes* provide the one-, two-, or three-dimensional angular velocities of the body segment to which they are attached. The design typically relies on the Coriolis effect whereby a body moving freely in a rotating frame of reference experiences the Coriolis force acting perpendicular to the direction of applied motion and to the axis of rotation. Segment orientations and joint angles can be determined by integration of the resulting angular rates if initial values are known and measurement biases are removed. However, the biases of MEMS-based gyroscopes are temperature-dependent and time-varying, which makes it difficult to estimate them during movements (Woodman, 2007).

To overcome the disadvantages of both sensor types, accelerometers and gyroscopes, *magnetometers* are often included in IMUs [also referred to as magneto inertial measurement units (MIMUs)] to improve orientation measurements, namely heading. Heading describes the angle of the sensor with respect to the horizontal direction of the magnetic north. In a magnetically undisturbed environment, magnetometers measure this component and a vertical component of the local earth's magnetic field. However, these readings are typically noisy and affected by magnetic disturbances originating from objects containing ferromagnetic material or emitting magnetic fields as usually the case in indoor environments (Schauer, 2017).

**Figure 3:**
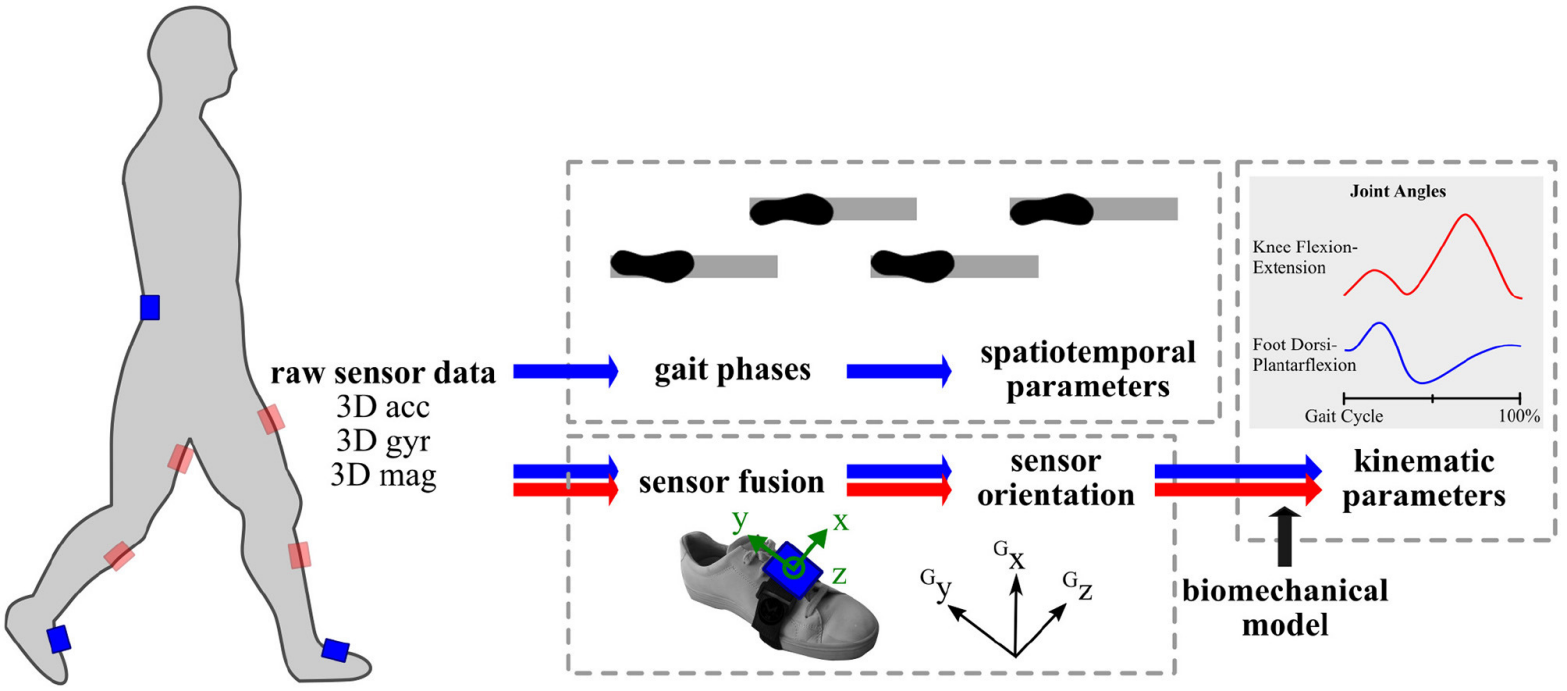
Methods overview for instrumented gait analysis with inertial sensors in commonly used positions on pelvis and lower limbs. acc, accelerometer readings; G, Global coordinate system; gyr, gyroscope readings; mag, magnetometer readings.

As the measurement signals of inertial sensors are well-known to be subject to errors such as time-variant sensor biases and measurement noise, reliable motion tracking requires computationally complex algorithms including state estimation methods and kinematic models (Seel et al., 2020). Three-dimensional strap-down integration and suitable sensor fusion algorithms combining the different signals are applied to estimate the real-time orientation of an attached IMU (Schauer, 2017), as illustrated in Figure 3. More precisely, the orientation of the inertial coordinate system, which is aligned with the housing of the sensor, is estimated with respect to a three-dimensional inertial reference coordinate system. The accuracy and precision of a wearable sensor system depend on how many sensors are used, where and how the sensors are mounted, and on the utilized algorithms. For example, the sensor coordinate systems should be sufficiently well-aligned with a meaningful coordinate system of the body part of interest, to which the sensor is attached, or methods for non-restrictive sensor-to-segment calibration or automatic anatomical calibration should be applied (Seel et al., 2014b).

For clinical gait data analysis, further mathematical tools are required to extract spatiotemporal gait parameters and often anatomical models are utilized for extracting kinematic parameters. Commonly in a two-stage approach, first gait events and phases are detected and, secondly, spatial parameters are determined. Various approaches exist on how and to what detail gait phases are detected from IMU recordings. Reliable gait detection can be achieved by exploiting the angular rates from the gyroscopes (Bertoli et al., 2018) or by combining them with the accelerometer measurements using peak detection algorithms (Mariani et al., 2013). Automated methods deploy adaptive thresholds based on the subject's walking style (Bejarano et al., 2014; Seel et al., 2014a). Spatial parameters are obtained by either signal integration, kinematic gait models, or ML techniques (Yang and Li, 2012; Caldas et al., 2017). Major gait parameters, for example, stride length, walking speed, can be derived with the most commonly used setup of two inertial sensors that are placed on the feet/shoes (e.g., Schlachetzki et al., 2017) or on the shank. Especially when postural control and balance parameters shall be extracted, a third sensor is added either on the chest, pelvis or lumbar spine and the acceleration is used to calculate COM and sway parameters (Mancini et al., 2012b; Curtze et al., 2015; Hsieh and Sosnoff, 2021). Recently, it has been shown that several gait events can be obtained from a single IMU at the pelvis even in individuals with neurological conditions (Pham et al., 2017). Tracking of the lower body or full body motion tracking is facilitated by further sensors (e.g., Schepers et al., 2018; Teufl et al., 2019).

Available commercial systems for gait and balance analysis applied in neurological patients are, for example, *Xsens MVN* (Xsens Technologies B.V., Enschede, Netherlands) for full-body motion tracking with up to 17 sensors, *Mobility Lab* (APDM, Inc., Portland, OR, USA) with six IMUs and data analysis software for different test scenarios (Dewey et al., 2014; Mancini and Horak, 2016; Morris et al., 2019), or *RehaGait* (HASOMED, Magdeburg, Germany) with up to seven inertial sensors and gait data analysis software (Donath et al., 2016). The sensors and systems diverge in their software, namely algorithms for sensor fusion and parameter estimation, as well as in their communication and housing. The housing and its dimensions vary depending on the battery capacities and on-sensor storage (Diaz et al., 2020). Furthermore, different data collection modes are available, such as real-time streaming or post-recording data download, and their transmission to a computer or smart device. The provided sampling rates correlate with the number of utilized sensors and show a large range (22–320 Hz), although this parameter has a high impact on the accuracy (Caldas et al., 2017). Besides, the pricing for IMU-based gait analysis systems varies strongly with individual wireless inertial sensor being available at affordable prices. However, the more detailed the gait analysis software has been evaluated, the more expensive it is. This is one reason for the trend toward own investigations and open-source gait analysis software (e.g., Gurchiek et al., 2019). Therefore, before deciding on a sensor system, the complete application scenario and budget should be outlined and recent developments need to be taken into account.

#### 3.1.2. Applications

**Inertial sensors in PD**. Inertial sensor technology applied in PD was able to reproduce the findings of distinct spatiotemporal gait characteristics including short steps, shuffling gait, and postural instability, specific for different disease stages and levels of motor impairment (Schlachetzki et al., 2017); cf. Section 2.3. A typical application is the instrumentalization of established clinical tests with IMUs with the goal of making the assessment rater-independent and gaining additional information (Palmerini et al., 2013). For example, Dewey et al. (2014) used the *Mobility Lab* system in an instrumented TUG and instrumented sway assessment in 135 PD subjects and 66 age-matched controls. For both tests, they identified multiple variables (e.g., stride length, turn duration, total sway area) that correlate with PD severity measures and differentiate PD subjects from controls. Instrumented gait analysis with IMUs offers the possibility to examine the differential effects of established and novel PD treatments on gait. A selection of exemplary studies is presented in Table 2.

**Table 2:** Exemplary clinical studies utilizing IMUs for gait assessment in PD.

**Publication**	**Study population**	**IMU position(s)**	**Clinical intervention**	**Outcome**
**IMUs for therapeutic outcome**				
Curtze et al. (2015)	*n* = 104 PD patients, *n* = 64 age-matched controls	Ankles, wrists, lumbar spine, sternum	Levodopa treatment (ON- vs. OFF-state)	Improved pace-related gait measures in ON-state: increased stride velocity and stride length, improved lower leg ROM and arm swing; impaired balance measures in ON-state: increased postural sway
Iijima et al. (2017)	*n* = 14 PD patients	Waist	Selegiline Treatment (before vs. after the addition/increase in dose)	Increased amplitudes and range of gait accelerations after dosage addition/increase in 40–63% of the patients; diminished fluctuations in gait throughout the day (86%)
Cebi et al. (2020)	*n* = 13 PD+FoG, *n* = 5 PD-FoG	Ankles, lumbar spine	DBS-STN (DBS-ON vs. DBS-OFF)	Reduced time to complete walking task, increased stride length, improved lower leg ROM; reduced freezing events (freezer subgroup)
**IMUs for cueing**				
Mazilu et al. (2015)	*n* = 9 PD patients	Feet, ankles, thighs, lumbar spine, wrists	Adaptive auditory cueing (metronome beats)	Trend toward reduced number of FoG episodes
Sijobert et al. (2016)	*n* = 13 PD patients	Foot	Gait-synchronized sensory electrical stimulation	Reduction of FoG events and reduced time to complete a walking task
Ginis et al. (2016)	*n* = 40 PD patients	Feet, ankles	Adaptive auditory feedback, personalized gait advice (active control)	Improved single / dual task gait speed (both groups), improved balance and quality of life (adaptive auditory feedback)
Ginis et al. (2017)	*n* = 28 PD patients, *n* = 13 age-matched controls	Feet, ankles, lumbar spine, wrists	Adaptive auditory feedback, continuous auditory cueing, adaptive auditory cueing (metronome beats)	Reduced deviation of cadence (continuous and adaptive cueing), maintaining cadence but increased fatigue (adaptive feedback)
Mancini et al. (2018)	*n* = 25 PD+FoG, *n* = 18 PD-FoG	Feet, shins, lumbar spine, sternum	Gait-synchronized tactile feedback at wrist, rhythmic auditory cueing	Both modalities reduced FoG during turning, increased smoothness of turns, decreased turning speed
Fino and Mancini (2020)	*n* = 43 PD patients	Feet, ankles, lumbar spine, sternum, wrists	Gait-synchronized tactile feedback wrist, rhythmic auditory cueing	Improved trunk stability (tactile cueing), but reductions in gait speed and stride length and increased stride time
Schlenstedt et al. (2020)	*n* = 36 PD+FoG, *n* = 18 PD-FoG patients	Shins, lumbar spine	Gait-synchronized tactile feedback wrist	Increased first step duration, no effect on anticipatory postural adjustments

For instance, Curtze et al. (2015) studied the effect of levodopa treatment on gait in a large cohort of patients and found that pace-related gait measures responded well to levodopa treatment, while balance parameters did not improve in the ON- compared to the OFF-state. Iijima et al. (2017) used 24 h single-accelerometer measurements from the trunk in order to track improvements in the gait fluctuations of PD patients after the addition/increase in dose of selegiline, showing a higher sensitivity than clinical scores. Recently, Cebi et al. (2020) used gait kinematics derived from IMU sensors placed at the hip and ankles to examine the therapeutic outcome of deep brain stimulation of the Nucleus subthalamicus (STN-DBS) on gait disorders in PD. Time to complete a 7 m walking task and number of steps were reduced and gait kinematics improved (stride length, ROM) 8 weeks after STN-DBS surgery in the DBS-ON compared to the DBS-OFF condition. In addition, freezers with a pre-surgical levodopa response of gait kinematics responded better to STN-DBS, indicating that the assessment with IMUss might be useful to predict the outcome of such treatments in specific patient subgroups.

Another popular application of IMUs in PD is as a tool to recognize and quantify FoG, a symptom which is rarely observed during clinical consultations since it occurs episodically. Freezing of gait usually appears in everyday life situations, i.e., during turning or walking through narrow doorways, and is associated with an increased risk of falls (Gray and Hildebrand, 2000; Bloem et al., 2004). Various sensor-based methods have been developed to objectively measure FoG in terms of number of episodes and episode duration (Moore et al., 2008; Rodrguez-Martn et al., 2017; Silva de Lima et al., 2017; Suppa et al., 2017; Pardoel et al., 2019). Sensor-based FoG detection opens up the possibility of monitoring FoG in the home environment of patients, which could facilitate the diagnosis and treatment of FoG (Suppa et al., 2017; Mancini et al., 2021).

Furthermore, IMUs are integrated in novel therapeutic cueing devices, which aim to monitor and treat gait disorders in PD. Cueing was shown to be effective in improving gait function in PD and a multitude of cueing paradigms exists (Muthukrishnan et al., 2019). Table 2 includes examples of cueing devices using IMUs to either administer gait-synchronized cues or to analyze the gait pattern in response to treatment. For example, the gait training tool *CuPiD-system* consists of wearable IMUs, a smartphone, and headphones to deliver intelligent auditory feedback on gait (Casamassima et al., 2014; Ferrari et al., 2016). Patients using the device showed improvements in maintaining cadence during prolonged walking, improved balance, and quality of life (Ginis et al., 2016, 2017). The *GaitAssist* system applies adaptive, rhythmic auditory cues and was used in the home environment of PD patients, who showed a trend toward reduced FoG episodes after several days of gait training with the system (Mazilu et al., 2015). Other cueing systems administer gait-synchronized sensory stimulation, which process IMU data online to analyze the gait while walking: Mancini and colleagues examined the effect of vibrotactile cueing at the wrist (*VibroGait*) and found reduced FoG, improved turning and trunk stability, increased first step duration, but reductions in gait speed and stride length (Harrington et al., 2016; Mancini et al., 2018; Fino and Mancini, 2020; Schlenstedt et al., 2020). Sijobert et al. (2016) developed a smart cueing device applying sensory, electrical stimulation at the lower leg via skin electrodes and found that the time to complete a walking task and the number of FoG episodes decreased.

These studies show that sensor-based gait measurements (1) might help to objectively examine treatment effects on gait disorders, (2) might facilitate the monitoring of treatment outcomes over longer follow-up periods, (3) may be used to predict the outcome of treatments in specific patient subgroups, and (4) could become integral part of new therapeutic methods.

**Inertial sensors in MS**. Shah et al. (2020) postulated that daily life monitoring with IMUs might be more sensitive to impairments from neurological diseases than laboratory IMU-based gait measures but that the analyzed neurological diseases (PD and MS) might require different gait outcome measures. Trunk-, shank-, or foot-placed IMUs have been frequently applied to measure gait and, especially, balance dysfunction in MS patients, commonly in the form of the instrumented TUG test (Shanahan et al., 2018): Spain et al. (2014) utilized IMUs to differentiate between mild MS, moderate MS, and control groups based on the variability in gait velocity, trunk motion, and sway (range, area). Craig et al. (2017) showed the reproducibility of the instrumented TUG results over two sessions and that stride velocity, cadence, and cycle time correlate significantly with disease severity and number of recent falls. IMU-based analysis has been found useful to detect even early changes in gait and balance in MS (Spain et al., 2012). Measurements with IMUs were able to reflect intra-individual changes in identified biomarkers associated with a change in clinical severity scores in a 12-month prospective study by Galea et al. (2017). Therefore, objective gait analysis with IMUs might increase the sensitivity of clinical and performance tests to monitor gait dysfunction in MS (Vienne-Jumeau et al., 2020). Moreover, spatiotemporal parameters from walking have been used to objectively measure MS disease specific characteristics, such as muscle fatigue, which could be helpful in monitoring and evaluating rehabilitation and treatment efficacy (Motta et al., 2016; Ibrahim et al., 2020). In the area of therapeutic aids and home-care, IMU-based fall detection is an emerging application for various gait disorders (Wang et al., 2020).

### 3.2. Smart Devices

#### 3.2.1. Technology

Although smart devices, such as smartphones or smartwatches, use inertial sensors as a technique, they are presented separately in this section due to their high presence and popularity in everyday life that makes them particularly interesting for long-term monitoring in home environments. Smart devices can be used as single sensor units like previously described IMUs, for example, by wearing a smart device on the hip for a postural control assessment (Kosse et al., 2015). The data can then be transferred and processed in the same way as previously described for IMUs. Usually, measurement setups are limited to two measurement locations, for example, one smartphone and a paired smartwatch. However, most frequently only one device is utilized (e.g., Chomiak et al., 2019; Hsieh et al., 2019). Available sampling rates for smart-device-based IMUs depend on the hardware (e.g., for Apple products^3^ (Cupertina, CA, USA), it is supposed to be at least 100 Hz), and can limit the range of applications. The reliability of smart device measurements for motion tracking is still being investigated (Vohralik et al., 2015).

In addition to regular IMUs, smart devices come with an integrated interface and specific software (“apps”) facilitating a user-friendly operation of the data assessment. Different apps provide different data collection modes, such as real-time streaming, recording, and post-recording wireless data download. In literature, mostly customized apps were used to record the sensor data and calculate gait or balance parameters on- or offline (Franco et al., 2012; Kosse et al., 2015; Chomiak et al., 2019), or to upload the data to cloud servers for offline gait analysis (Manor et al., 2018). As an advantage to standalone IMUs, smart devices promote direct biofeedback in the form of visual, auditory, or haptic signals. Due to these features, smartphones are often combined with IMUs for gait monitoring and therapy systems (e.g., Ferrari et al., 2016; Palmerini et al., 2017).

#### 3.2.2. Applications

Although the use of smart devices to assess gait and balance is under extensive investigation, most applications are still under development. Multiple studies explore smart devices for balance assessments measuring trunk movements and postural stability but so far mostly in neurologically intact participants (e.g., Alberts et al., 2015a,b; Kosse et al., 2015; Hsieh et al., 2019). Roeing et al. (2017) reviewed 13 studies and found that five evaluated the validity of their smartphone applications for balance and risk of falls assessment; the results demonstrated strong concurrent validity with standalone accelerometry, 3D motion capture, and force plate measurements. Three of these studies included a measure of reliability revealing high ICC values for mixed variables (Mellone et al., 2012; Cerrito et al., 2015; Kosse et al., 2015; Roeing et al., 2017). Standardized clinical assessments, such as sit-to-stand evaluation (Cerrito et al., 2015; Marques et al., 2021), the TUG (Mellone et al., 2012; Ponciano et al., 2020), and postural balance (Hsieh and Sosnoff, 2021), were instrumented using a single smart device. Also applications in rehabilitation in the form of biofeedback loops with potential use at home, e.g., as a smartphone-based audio-biofeedback in order to improve balance during bipedal standing (Franco et al., 2012), are being evaluated. However, special research interest is on utilizing smart devices for gait assessment as there lies a great potential for long-term monitoring in everyday activities. Fall detection with smart devices is already available on the market in the form of *Apple Watch Series 4–6* (Apple Inc, 2020). The accelerometer and gyroscope readings from the wrist are used in combination with a fall detection threshold yielding a high false-positive rate (Wang et al., 2020).

Most approaches aim at extracting gait parameters from the use of a single smart device (Ellis et al., 2015; Kosse et al., 2015; Manor et al., 2018). For example, Manor et al. (2018) created an app for systematic gait data recording and analysis that can be performed independently by the user either in the laboratory or at home with the smartphone placed in the trousers' front pocket. When comparing normal and dual-task trials in neurologically intact volunteers, average stride times derived from the app demonstrated high correlation with the simultaneously used instrumented mat in the laboratory. Lipsmeier et al. (2018) explored the potential of smartphones for assessing biomarkers in PD that might serve as outcome measures in clinical trials. The authors presented moderate to strong retest reliability and successful discrimination between PD and controls with increased sensitivity compared to traditional clinical scales (Buckley et al., 2019).

Despite regular gait assessment, smart devices were evaluated for continuous monitoring in PD for FoG detection and fall prevention. For example, Ellis et al. (2015) developed a mobile application with the smartphone at the front waist to track gait and its variability, an indicator for FoG in PD, presenting it as an alternative to conventional gait analysis technologies. Chomiak et al. (2019) utilized an *iPod Touch*, worn on the thigh, and ML to identify gait-cycle breakdown and freezing episodes of varying duration. Ahn et al. (2017) presented a system for FoG detection and visual cueing based on smart glasses (Android): The subject's movements are tracked using the inertial sensor from the glasses, which projects visual patterns in the case of a recognized FoG event. Furthermore, numerous smartphone applications have been designed for the assessment and monitoring of multiple health parameters in patients with PD (Monje et al., 2019). The apps combine questionnaires, cognitive, voice, and motor tasks providing repeated measures of the patients motor state along with valid and clinically meaningful knowledge of symptom evolution (Bot et al., 2016; Lakshminarayana et al., 2017; Lipsmeier et al., 2018). Similar applications are available for monitoring MS patients [e.g., elevateMS by Pratap et al., 2020].

These diverse applications of smart devices in gait and balance assessments reveal their future potential to be utilized for objective evaluation of treatments over short and long follow-up periods, closed-loop applications, and telemedical consulting in real-life environments.

### 3.3. Instrumented Insoles

#### 3.3.1. Technology

Instrumented insoles are insoles that have integrated force or pressure sensors to measure changes in pressure between the foot and the ground. Force sensors measure the applied force discriminating the component of each axis that is measured, whereas pressure sensors are non-discriminating and thereby measure the combined ground reaction force (de-la Herran et al., 2014). Most commonly used insole sensors are capacitive, resistive piezoelectric, and piezoresistive sensors (de-la Herran et al., 2014). The measurement principle is based on the detection of voltage changes caused by fluctuations in electrical capacity or electrical resistance in semiconductor materials due to stretching or compression (Chen and Yan, 2020). The choice of sensor depends on the desired range of pressure/force, sampling rate, and sensitivity (Diaz et al., 2020). Insoles typically incorporate arrays of sensors measuring a spatial pressure/force profile over the plantar foot surface (Shanahan et al., 2018). The profile varies during the gait cycle and depends on a person's body weight: In healthy gait, the maximum vertical force is applied and, thereby, the maximum pressure occurs when the whole body weight is on one leg/foot during the stance phase (Clarke, 1980). No force is applied during the swing phase. The profile's spatial resolution depends on the number of integrated sensors in the insole, its temporal resolution on the applied sampling rate, and its sensitivity on the utilized sensor and analog-to-digital converter.

Available systems are among others the *F-Scan* (Tekscan Inc., Boston, MA, USA) with 3.9 force-sensitive resistors per cm^2^, the *Moticon SCIENCE* pressure insoles (Moticon, Munich, Germany) with 16 capacitive pressure sensors, or *WalkinSense* (Kinematix SA, Sheffield, UK) with eight force-sensing piezoresistors. The latter two and other newer insole types often incorporate additional sensors such as an IMU (Arafsha et al., 2018). Besides, the available systems differ in the type of power supply, data transmission and storage, in the user operation, and associated analysis software.

The validity of discrete pressure and force measurements with insoles is comparable to optical motion capture and they show a high reliability within and between trials (Shanahan et al., 2018). From the profiles, spatiotemporal gait parameters (e.g., stride time, gait phases) can be extracted. However, patients with neurological gait disorders tend to walk slowly, shuffle, and perform short and dragged steps making it challenging for automatic gait event detection based on heel strike or initial contact (Pirker and Katzenschlager, 2017; Diaz et al., 2020). For balance analysis, insoles are regularly used to measure the COP to evaluate postural stability (Ma et al., 2016).

#### 3.3.2. Applications

So far, the clinical application of instrumented insoles in PD patients has mostly been limited to the differentiation between PD and controls. Extracted gait and balance parameters have been used successfully for discrimination between the groups (Mazumder et al., 2018; Chatzaki et al., 2021), in line with the findings from established laboratory gait assessments. Furthermore, instrumented insoles have been investigated for their ability to recognize and quantify FoG in PD (Popovic et al., 2010; Shalin et al., 2020; Pardoel et al., 2021). Pardoel et al. (2021) combined features derived from a pressure-sensing insole and IMUs on the leg to detect FoG in 11 PD patients. The authors reported that the combination of both modalities outperformed classification models that used data from a single sensor type. In a small data sample (*n* = 5), Shalin et al. (2020) demonstrated that foot pressure distributions from 60 × 21 sensor-arrays could be used for FoG prediction (0.5–3 s before FoG onset). Therefore, together with inertial sensors, instrumented insoles could be integrated into therapeutic cueing devices for treating gait disorders in PD (cf. section 3.1).

Few studies utilized insoles to examine gait function in MS patients so far (Shanahan et al., 2018). Viqueira Villarejo et al. (2014) reported an increased plantar pressure during the stance phase and variability in step timing in MS compared to controls. Galea et al. (2017) quantified MS-related gait and balance deterioration over 12 months using EMG and insoles and observed decreases in gait speed and balance scores, and an increase in double support time. Doḿınguez et al. (2020) validated gait velocity and other parameters from a new insole system with an incorporated IMU against a common instrumented walkway in 205 MS patients. The results revealed a high correlation between devices in velocity, ambulation time, cadence, and stride length. Note that spatial parameters, such as stride length and stride wide, can only be derived from the IMU data (Farid et al., 2021).

Although the use of insoles is unobtrusive and, therefore, has a high potential in monitoring daily activities (Diaz et al., 2020), the hesitant use in research and clinical application may have practical reasons. For reliable measurements, diverse sole sizes must be available to cover the variety in foot sizes. Systems with multiple soles and validated analysis software can initially require a five-digit amount. Due to the mechanical stress during walking, the soles' life is limited. People must wear shoes that allow the use of additional insoles. Furthermore, shoes must be taken off and put on again to set up the measurement, an additional obstacle for elderly patient groups such as PD. However, for long-term monitoring in the future, the ease of integration in patients' everyday life could be an advantage.

### 3.4. Summary and Discussion

Wearable sensor technology is currently being applied and explored in existing and newly developed clinical gait and balance assessments as well as for long-term monitoring of various activities in daily living. For multiple reasons, body-worn sensors are of great value for balance and gait assessments in neurological disorders: Their high level of portability theoretically facilitates unlimited use in laboratory research environments, clinical settings, and home environments. No line-of-sight restrictions apply as in vision-based technologies. The small, lightweight, and wireless devices do not restrict the subject's movement. In contrast to laboratory-based methods, wearable devices might come at low prices and facilitate easier setups. When provided with a graphical user interface and validated analysis software, usability can be as good that patients can record data on their own. The number of gait parameters that can be extracted from wearables has expanded dramatically over the last years and new, more robust algorithms are under permanent development.

However, all these potential advantages are not always met in the available systems. The gait estimation algorithms for IMUs and insoles are often still in exploration, not evaluated to a reliable extent in the desired target group. The optimal sensor layout is still debated and requires a trade-off between usability and accuracy. Furthermore, it is challenging to calculate paths and distances traveled (Buckley et al., 2019). Necessary sensor-to-segment alignment, the need for precise manual sensor attachment, and required calibration movements by many methods halt the advance of inertial sensor techniques into clinical trials. When utilizing magnetometer readings, measurement errors occur in magnetically-disturbed environments, such as typical clinic or home environments containing electronic devices and objects of ferromagnetic material (de Vries et al., 2009). With increasing algorithm complexity, required processing resources rise yielding high energy consumption and waiting times between subsequent recordings. Still, algorithm development is an active area of research tackling these issues (e.g., Marín et al., 2020; Laidig et al., 2021). Also, existing hardware issues, such as limited recording time by battery and storage capacity, and data loss during the wireless transfer from sensors to applications or cloud servers should be a trivial problem in the future. Patient user interfaces continue to improve (Shanahan et al., 2018).

In addition to their use in recording and analyzing gait and balance disorders, wearables can be applied in rehabilitation technologies or therapeutic aids, such as a cueing device to treat gait impairments in PD (cf. Table 2). Besides beneficial therapeutic effects, full-time body-worn sensors allow long-term monitoring and might contribute to the individualization of therapies as well as to telemedicine concepts. The objective tracking and quantification of a PD patient's motor activity over the day is valuable information for the precise adjustment of individual medication plans. Particularly in times of global pandemics, where the number of regular face-to-face visits is reduced (Roy et al., 2020), automatically extracted and shared parameters from wearables have the potential to support clinical decisions. Automatic evaluation methods of data from wearables in clinical gait and balance assessments (e.g., Karatsidis et al., 2017; Nguyen et al., 2019) but also in unrestricted activities of daily living (e.g., Roth et al., 2021) are constantly investigated. Machine learning techniques are the driving force behind this rapid growth of applications. Still, a remaining challenge lies in obtaining validated measures and standardized motor parameters that predict relevant clinical outcomes for each neurological disease (Monje et al., 2019; Shah et al., 2020). Further investigations are required before the analysis of locomotion in everyday activities becomes reliable and thereby clinically relevant (Graham et al., 2008; Lord et al., 2013).

## 4. Non-Wearable Technologies: Vision-based Motion Analysis

Marker-less optical motion capture systems have become popular with the launch of affordable in-depth cameras. Even though computer gaming and virtual reality serve as the main drivers for this rapid evolution of digital image processing, the practical application in the health sector has been discussed and performed frequently (Clark et al., 2012; Albert et al., 2020). Despite being an older technology, motion tracking with optical markers is generally handled as the gold standard with which newly developed technologies for gait analysis are compared. However, marker-based tracking requires an extensive, expensive, non-portable setup. Similar restrictions apply for instrumented mats, walkways, or treadmills, although they are portable on a large scale and often easier to handle. For the sake of completeness, we mention these approaches as references; overviews of gold standard methods can be found elsewhere (e.g., Shanahan et al., 2018; Celik et al., 2021).

Presently, vision-based non-wearable technologies with standard cameras or depth cameras are increasingly applied in human motion tracking. Image acquisition is most conveniently achieved via standard 2D cameras that output images as 2D pixel grids. Each pixel traditionally carries a red, green, and blue (RGB) value, with intensities ranging from 0 to 255. However, 2D cameras do not provide any spatial depth information on the tracked pose. This information has to be obtained either by performing additional post-capture processing with machine learning (ML) algorithms, by using multiple cameras, or by switching to another technology, such as depth cameras which provide 4D information on the tracked object. Pixels obtained with depth cameras are primarily coupled to the distance of the tracked object from the sensor and are typically paired together with a classical RGB value. Both, standard and depth cameras, are able to extract detailed information required for biomechanical analyses.

### 4.1. In-depth Camera Technology

**Figure 4:**
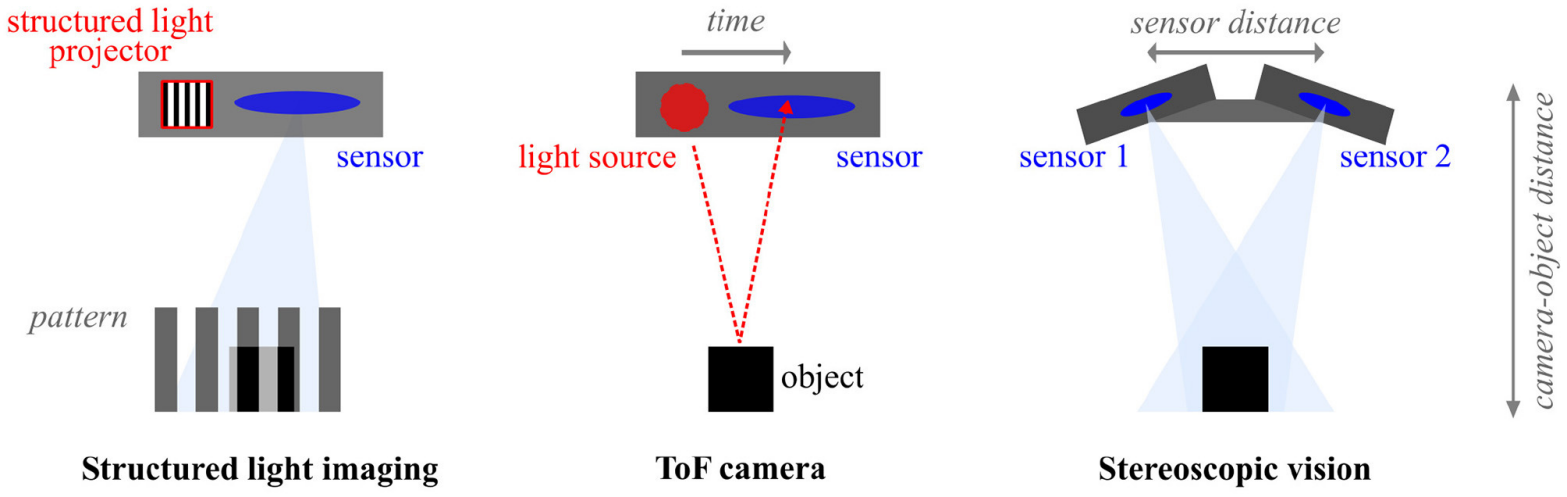
Illustration of different camera technologies for in-depth measurements.

There are several types of in-depth cameras that rely on different methods to infer depth, as illustrated in Figure 4. Structured light imaging uses patterned light to capture the 3D topography of a surface (Geng, 2011). Here, the scale and direction of a distorted pattern are used to assess the depth of an object. Furthermore, time-of-flight (ToF) technology measures the time it takes for infrared light to travel toward an object and reflect into the imaging sensor (Kolb et al., 2010). The corresponding phase shift in the signal is subsequently measured and converted into distance. Lastly, stereoscopic vision (also: stereotactic imaging) incorporates two or more stereo cameras to compare two or more simultaneously recorded images for the estimation of depth. Likewise to human eyes, the distance between the cameras is fixed and used to measure the closeness of an object on multiple juxtaposed images obtained by using any type of light.

Two well-known, affordable depth-sensing cameras that are frequently used in medical applications are *Kinect* (Microsoft, Redmond, WA, USA) and *RealSense* (Intel, Santa Clara, CA, USA), which are reviewed here as examples due to their manifold occurrence in literature on gait and balance. Other commercially available systems on the market are, e.g., *ZED* (StereoLabs, San Francisco, CA, USA) or *XtionPro* (ASUS, Taipeh Taiwan).

#### 4.1.1. Microsoft Kinect

Microsoft's *Kinect* is a motion-sensing device originally developed for gaming purposes and is one of the earliest motion capture technologies of its kind to be used in medical research. Owing to *Kinect's* long history in pose estimation, it has been well-assessed in various research settings and utilized in tracking different movement patterns. Throughout the last decade, several versions of *Kinect* have been produced: The introductory model *Kinect 1* (2010) integrates a structured near-infrared light source with an accompanying sensor to capture the reflected light patterns, whereas *Kinect 2* (2013) and *Azure Kinect* (2019) use wide angle ToF cameras (Zhang, 2012).

Although *Kinect 1* is a well-established system, when compared to gold standard techniques, it provides only basic motion capture capabilities such as collecting temporal gait parameters, estimating single joint angles, or assessing postural control during reaching and balance tasks (Clark et al., 2012; Schmitz et al., 2014). In kinematic gait recordings, the system generally underestimates joint flexion and overestimates extension during walking in the sagittal plane. Here, stride timing measurements perform surprisingly well with the highest accuracy at low gait speeds, despite a high error in hip and knee displacement (Pfister et al., 2014). Clothing and different body shapes were discussed as possible reasons for measurement errors. Therefore, approaches that use additional multi-layer filtering, where the estimated pose is further refined through a synthetic library of posture variations, can alleviate some tracking inaccuracies, increase parameter precision, and allow for better recognition of occluded body parts (Shotton et al., 2011; Wei et al., 2012; Xu et al., 2013). Moreover, recording frameworks with multiple cameras were able to improve pose estimation accuracy and approximation of occluded segments at the costs of a higher setup effort (Gao et al., 2015).

Nonetheless, *Kinect 1* has been used in several clinically-oriented studies to measure lower body biomechanics for determining stride time, length, and speed in healthy individuals (Gabel et al., 2012; Auvinet et al., 2015). In the context of PD, scientists used wavelet-based digital signal processing to analyze gait parameters and quantitatively distinguish gait phases with an accuracy of up to 93% (Muñoz et al., 2018). Spatiotemporal parameters were distinguishable in stage II and III PD patients compared to a control group, reaching a maximum accuracy of 97.2% after classification with a neural network (NN) (Ťupa et al., 2015). Likewise, *Kinect 1* technology has been implemented in MS gait analysis to discern MS patients from neurologically intact controls by differences in the average walking speed and lateral body sway (Behrens et al., 2014), or ROM, stride length, and step width (Gholami et al., 2016).

The newer *Kinect 2* system uses continuous-wave ToF technology instead of structured light, enabling a more stable data feed with an increased accuracy within the measurement range of 4m (Gonzalez-Jorge et al., 2015). In clinical assessments, *Kinect 2* displayed an adequate performance when tracking joint center displacement (Napoli et al., 2017). The second generation demonstrates better accuracy in joint estimation and stays more robust to body rotation as well as occlusions during various movements like walking and jogging (Wang et al., 2015; Guess et al., 2017). Therefore, *Kinect 2* seems to outperform *Kinect 1* in locomotion tracking except for foot position tracking during standing, where a larger amount of noise is generated, possibly due to ToF artifacts (Otte et al., 2016). *Kinect 2* reliably assessed spatiotemporal parameters during comfortable and fast-paced gait (Mentiplay et al., 2015). However, significant performance variations in different motion planes and incompatibility with certain functional movements still exist in *Kinect 2* when compared to marker-based systems, especially in the context of compound joint movement (Mentiplay et al., 2015). The validity of lower limb joint kinematics depends on the camera's capture angle for recording the walking subject. Moreover, in treadmill walking, accuracy levels appear to vary across gait parameters, with temporal parameters based on heel strike having fewer errors than those based on toe-off, and their accuracy fluctuates with changing walking speeds (Xu et al., 2015). Linear pelvic and trunk ROM can still be tracked with reasonable precision at 70 and 90% of maximal locomotion speed, providing a reliable reference point across all velocities (Macpherson et al., 2016). In attempts to use multiple *Kinect 2* cameras to achieve a higher tracking accuracy, several cameras have to be calibrated together via geometric trilateration. The distance between the subject and at least three recording cameras is measured through signal strength. When used to determine gait parameters, three *Kinect 2* sensors show a much higher spatiotemporal reliability compared to a single *Kinect 2* camera (Yang et al., 2016).

*Kinect 2* has been applied in PD patients, where 92% of freezing episodes, 91% of tremor occurrences, and 99% of falling incidents could be detected with customized algorithms (Bigy et al., 2015). Moreover, *Kinect 2* measurements in combination with customized algorithms were able to consistently produce results similar to a marker-based system and output significant differences between PD and control groups for stride length, gait, and swing velocity (Eltoukhy et al., 2017; Sabo et al., 2020). In MS patients, moderate and fast walking speed measurements agree with results derived from marker-based systems, but once more, only if combined with customized software or auxiliary ML-based classifiers (Bethoux et al., 2018; Elkurdi et al., 2018). Indeed, it seems that additional ML algorithms or NNs can frequently increase the validity and reliability with depth cameras (Rocha et al., 2018).

Microsoft's *Azure* is the most recent *Kinect* upgrade that supports additional features and several depth-sensing modes. Although part of the same production line, it has been specifically designed for distinctive non-gaming purposes such as research and health care use. Compared to *Kinect 2, Azure* has a higher angular resolution, lower noise, and better tracking accuracy (Tölgyessy et al., 2021). When used as a dual system consisting of two cameras, *Azure* outputs precise knee angles and demonstrates an overall improved validity over *Kinect 2* (Ma et al., 2020b). During the estimation of sagittal hip and knee joint angles, a single *Azure* appears to have a superior depth resolution and shows better tracking performance when subjects walk at non-frontal camera viewing angles (Yeung et al., 2021). In treadmill walking, spatial gait parameters (e.g., step length and width) can be measured more reliably with *Azure*, though the accuracy of temporal parameters (e.g., stride duration) does not change significantly between the two models. Interestingly, *Kinect 2* seems to outperform *Azure* regarding upper body tracking. However, an overall increase in the quality of lower extremity parameters and the additional introduction of integrated deep learning-based body tracking algorithms create appeal for *Azure* to be used in gait rehabilitation (Albert et al., 2020). As far as the application of *Azure* in PD and MS studies is concerned, to our knowledge there has not been any material published yet.

#### 4.1.2. Intel RealSense

Intel's *RealSense* cameras stem from several generations of stereo depth cameras with a production start in 2015. The system comprises a left-right depth stereo camera pair and an additional color camera. The stereo cameras use textured light to ensure unambiguous image matching, which in turn enables more accurate depth measurements. Accordingly, stereotactic systems including *RealSense* are less sensitive to noise compared to other in-depth camera types, which allows for a more flexible experimental setup (Keselman et al., 2017; Zabatani et al., 2019). In motion analysis, the system can be used to measure a definite amount of spatiotemporal variables, however, joints with multiple degrees of freedom exhibit inaccuracies yielding difficulties for the forthcoming gait data analysis (Mejia-Trujillo et al., 2019). In general, *RealSense* seems to perform better at slow to normal walking speeds located in small to medium-sized environments (Hausamann et al., 2021). Auxiliary tools can be used to extend the current three-part system to up to six cameras to improve body shape and joint position tracking (Boppana and Anderson, 2019). Intriguingly, despite having an older production age, both *Kinect 1* and *2* seem to rival *RealSense*'s signal quality and capture range during walking (Mejia-Trujillo et al., 2019). Moreover, temporal parameters seem to exhibit slightly better accuracy when recorded with *RealSense*, whereas spatial parameters retain similar values to *Kinect* measurements (Gutta et al., 2021). While a few gait studies with Intel's *RealSense* exist, up to the present moment no publications known to the authors have used the technology to assess gait and balance explicitly in either PD or MS patient groups. However, *RealSense* convincingly holds the potential to be used in clinical research, whether as a new method for motion tracking or as a *Kinect* substitute (Clark et al., 2019).

### 4.2. Standard Camera Technology

In daily clinical practice, video recordings are still predominantly recorded with conventional standard cameras. Clinicians may film their patients during outpatient or inpatient visits (e.g., in frequented hallways) or are presented with home videos for neurological evaluation (Sato et al., 2019). However, the material has so far only been used for subjective assessment and documentation, not exploiting its full potential. The use of standard camera material for motion analysis would require a less demanding setup, fewer recording constraints, and would offer more favorable pricing, and integrability into daily research, clinical and telemedical settings. The resulting demand for swift algorithms that accurately determine body part locations on video images drove the development of numerous approaches for pose estimation in standard imaging. In comparison to depth cameras that output distance information without requiring training and often include built-in post-processing software, standard camera footage has to be analyzed offline by a separate learning pipeline for motion tracking. The software pipeline either determines pose coordinates in 2D or deduces depth, in case 3D coordinates are the desired output. In this section, we go over recent findings in pose estimation and discuss available toolboxes designed as ready-to-use software packages for a broader scientific audience that might be applied for gait analysis in neurological disorders.

#### 4.2.1. 2D Pose Estimation

Most recent 2D pose estimation approaches rely heavily on contemporary advances in deep learning, a branch of ML that employs NNs with many layers. With an annotated image data set where objects have been manually labeled, a NN can be trained via supervised learning to classify and track those objects. Pose estimation algorithms frequently use convolutional neural networks (CNNs) as their architectural foundation with multiple layers (e.g., Toshev and Szegedy, 2014). The greatest advantage of CNNs is their ability to learn feature representations directly from the data set in use, which removes the need for additional training data, thus, ensuring a straightforward experimental flow. Their convolutional structure produces 2D probability maps for the location of each body part after they had been trained to recognize image features that belong to specific shapes (e.g., knee, foot) (Wei et al., 2016). This establishes a statistical relationship between the input images and output pose key-points, which can be used to track pose in yet unanalyzed data and make predictions on the spatiotemporal appearance of tracked key-points.

**Figure 5:**
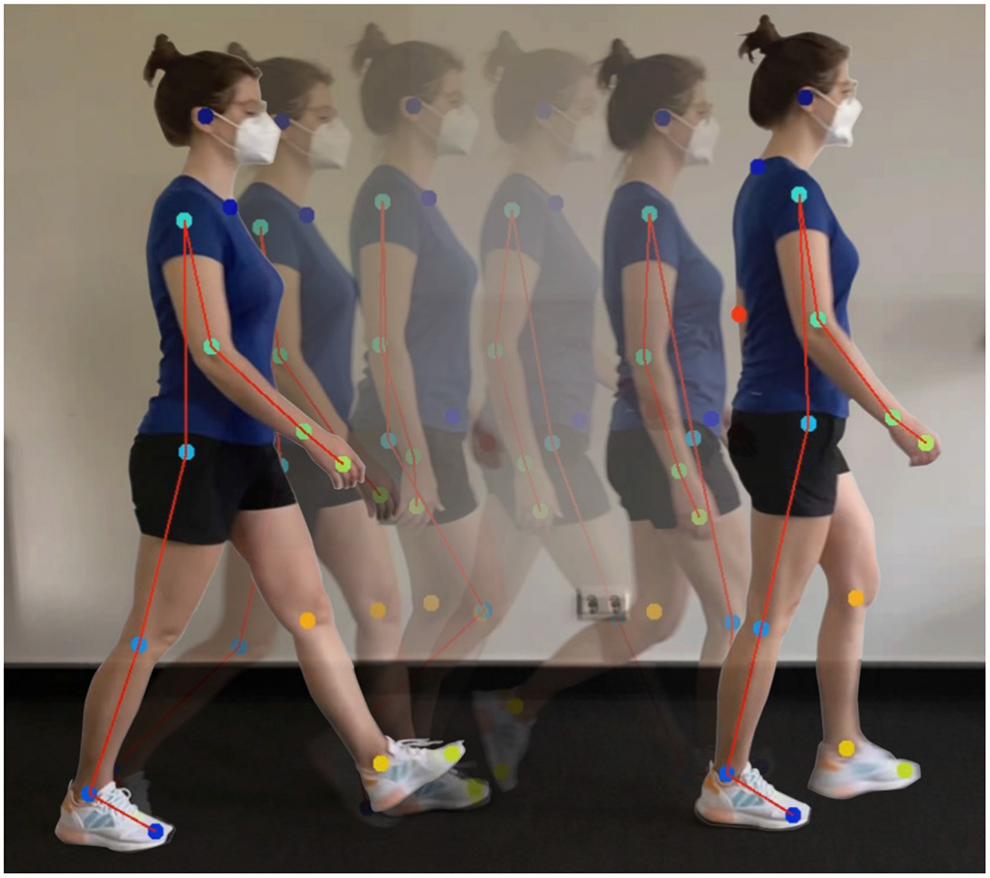
An example of 2D motion tracking performed with DeepLabCut. Here, several joints are being tracked simultaneously to determine the exact limb position during straight walking.

In video data, 2D pose tracking represents a unique set of challenges and numerous network designs have been created to optimize both for speed and reliability in their specific study context. Contrary to static image analysis, images that have been extracted from video frames are often subject to motion blur, frequent body occlusions, unconventional subject positions and further represent large data sets due to the sheer amount of frames in a single video (cf. Figure 5). The continuously increasing amount of new pose algorithms also evokes the demand for largely manually annotated data sets that thematically represent the defined area of research: sports, outdoors, medical research, and many others (Sigal et al., 2010; Ionescu et al., 2013; Andriluka et al., 2014). To withstand these challenges, attempts have been made to increase the quality of parameter supervision by, for example, cross-correlating features in adjacent video frames or integrating various mathematical approaches with NNs, and to reduce the amount of required pre-labeled data (Ouyang et al., 2014; Szegedy et al., 2015; Tompson et al., 2015; Feichtenhofer et al., 2017).

In gait analysis, established preliminary models use standard cameras ranging from simple mobile phone cameras to multiple cameras accompanied with additional sensors such as IMUs or floor sensors (Alharthi et al., 2019; Viswakumar et al., 2019; Vaith et al., 2020; Stenum et al., 2021). The validity, reliability, and processing time of these models vary according to the type and quality of camera footage, computational system architecture, type and amount of training data used as well as many other factors. Therefore, choosing a suitable pose estimation model is strongly influenced by the experimental setting and might depend on the number of tracked legs, frequency of body part occlusions, subjects' clothing, and room background color. Until these and other issues are resolved, 2D pose estimation will not be applied on a wide scale in the clinical field. However, first studies indicating possible applications of this technique in neurological disorders have been published: Li et al. (2018) combined the outcome of convolutional pose machines with ML-based classification for discriminating disease and symptom severity in PD patients in tasks such as toe-tapping and stamping. Hu et al. (2019) successfully established a novel graph CNN to classify freezing episodes from regular gait in the TUG test of 45 Parkinsonian patients, recorded in frontal view.

#### 4.2.2. Single-View 3D Pose Estimation

Occlusion of body parts has continuously presented a challenge to 2D human pose estimation for gait analysis, especially when both legs are tracked simultaneously, as desired, for example, for analyzing gait symmetry in PD. Thus, advances have been made toward setting the pose in a 3D coordinate framework instead of 2D by subsequently generating a 3D environment from images obtained by a single RGB camera. Complementary 3D pose libraries can be used to create 2D projections from virtual camera views. In such cases, 2D pose estimation is performed on input images and then depth is calculated using an additional pre-existing 3D library as a reference (Chen and Ramanan, 2017). However, the employment of 3D libraries requires even larger amounts of annotated data. To address this challenge, specialized CNNs have been implemented to output 2D key-points together with body silhouettes, which are later synchronized with a mathematically generated 3D body mesh model to estimate full 3D pose (Loper et al., 2015; Pavlakos et al., 2018). Moreover, some networks specialize in the detection of individual people from group images and automatically crop out single subjects that are present on the input image, subsequently performing individual 2D pose tracking and later placing the obtained parameters into a virtual 3D environment (Moon et al., 2019). Recently, single-view 3D pose estimation has been integrated into the gait analysis of PD patients, where spatiotemporal parameters including step length, velocity, and cadence evaluated with a deep learning pose estimation algorithm seemed in good agreement with reference data obtained through pressure sensors (ICC > 0.9) (Shin et al., 2021).

#### 4.2.3. Multi-View 3D Pose Estimation

The multi-view approach to 3D pose estimation is an alternative scheme that further reduces training set size and eliminates the need for large 3D libraries. One strategy is to train the network on images from multiple cameras before predicting the 3D pose from images obtained by a single camera (Rhodin et al., 2018). Specifically, a network is trained to predict the same 3D pose regardless of camera perspective and can perform 3D predictions solely based on 2D imagery. Other methods include algebraic and volumetric triangulation that are speculated to be more robust to occlusions or partial body visibility (Iskakov et al., 2019). Further strategies use so-called key-point coordinates instead of heatmaps (Pavllo et al., 2019) or employ a multi-stage architecture to reconstruct the 3D pose from 2D heatmap predictions at each CNN processing stage (Tome et al., 2018).

As multi-view 3D pose estimation approaches effectively deal with body part occlusions and simultaneously alleviate the need for large training libraries, they present a promising tool in gait tracking. Indeed, such models are able to qualitatively reproduce locomotion compared to marker-based motion capture, albeit still producing a small error rate in the final 3D pose (Nakano et al., 2020). Therefore, technical challenges of multi-view systems such as the setup of multiple cameras, triangulation, and more extensive processing make the experimental setting more demanding but at the same time offer an opportunity to improve the quality of gait parameters.

#### 4.2.4. Software Toolboxes

While numerous algorithms have been created in the attempt to improve the performance of 2D and 3D pose estimation algorithms, we will now briefly summarize several that have been pre-packaged as software toolboxes and are being used by a wider, non-specialist scientific community to track human motion promoting new fields of application (Table 3).

**Table 3:** Overview of available software toolboxes for 2D and 3D pose estimation from 2D cameras.

**Toolbox**	**Modality**	**Feature**	**Tracking**	**Gait analysis research**
*DeeperCut*	2D	ResNets, pairwise terms	Multiple	-
*DeepLabCut*	2D/3D	Pre-trained ResNets	Single*	Cronin et al., 2019
				Needham et al., 2021
*OpenPose*	2D/3D	Part Affinity Fields	Multiple	Xue et al., 2018
				Gu et al., 2018
				Viswakumar et al., 2019
				D'Antonio et al., 2020, 2021
				Zago et al., 2020
				Needham et al., 2021
				Stenum et al., 2021
*Anipose*	3D	Pre-trained ResNets	Single	-
*DeepPoseKit*	2D	Multi-scale inference	Single	-
*AlphaPose*	2D	Regional pose estimation	Multiple	Needham et al., 2021

*Designed for single person tracking, but can optionally perform multi-pose tracking.

One of the earliest of such packages is *DeeperCut*, a multi-person pose estimation method based on the integer linear programming approach *DeepCut* (Pishchulin et al., 2016). Here, deep residual neural networks (ResNets) have been adapted inside a convolutional architecture in form of a sliding window-based body part detection (He et al., 2016). ResNets build on constructs known from pyramidal cells in the cerebral cortex: They utilize skip connections, or shortcuts to jump over some network layers and map nonlinearities. Moreover, *DeeperCut* features image-conditioned pairwise terms or architecture components that indicate the presence of other body parts in the vicinity of a tracked point and group these body parts into a valid pose configuration (Insafutdinov et al., 2016). Published in 2018, *DeepLabCut* is a more recent tracking toolbox. Although a CNN architecture as well, *DeepLabCut* significantly differs from its predecessor *DeeperCut* by implementing pre-trained ResNets, which fine-tune the already existing node weights following the tracked body part. Therefore, *DeepLabCut* exhibits a faster performance and requires a smaller amount of pre-labeled images for training. After network processing, the user can readily access spatial coordinates and the existential probability of every tracked body part, stored in the form of x- and y-coordinates for each video time frame (Mathis and Warren, 2018; Mathis et al., 2018). Figure 5 shows an example of 2D motion tracking with *DeepLabCut*. Additional reconstruction of 3D kinematics with *DeepLabCut* is possible by either establishing individual networks for each camera view or training a single network that generalizes across all views (Nath et al., 2019).

*OpenPose* is a real-time 2D pose estimation approach developed for motion processing of multiple individuals on a single image. An integral part are Part Affinity Fields (PAFs), a set of 2D vector fields that encode the orientation and location of limbs on the analyzed image. Moreover, PAFs are bottom-up representations of unstructured pairwise relationships between detected body parts that enable the reconstruction of the full-body pose while decreasing the total computational cost. As with any multi-person tracking algorithm, *OpenPose* faces obstacles like subjects present on the image at different positions or scales and body part occlusions (Cao et al., 2019). *OpenPose* has recently been implemented with multiple synchronized cameras to evaluate motor performance in a 3D pose framework. Compared to a marker-based system, the mean absolute error of points tracked during walking equaled less than 30 mm, excluding 10% of cases where *OpenPose* initially failed to recognize the correct body segment during 2D estimation (Nakano et al., 2020).

Several other prominent pose estimation toolboxes exist which have not yet been frequently featured in gait research: *Anipose* is an open-source toolkit designed to augment the existing 2D tracking methods for accurate pose tracking in a 3D setting. It deploys optimization on the calibration, triangulation, and filtering over multiple camera views that accompanies the processing by antecedent NN packages (Karashchuk et al., 2020). *DeepPoseKit* aims to resolve the limitations of over-parametrization by pre-trained ResNets and the lack of robustness in GPU-based approaches. The pipeline is based on alternative confidence map processing methods, multi-scale inference, and GPU-oriented convolutional layers (Graving et al., 2019). Lastly, *AlphaPose* is another open-source multi-pose estimator featuring a regional multi-person pose estimation (RPME) framework (Fang et al., 2017). During training, the RPME pipeline detects single humans on the image by establishing bounding boxes around each individual. Afterwards, single pose estimation is performed on each bounding box and the output is further refined (Xiu et al., 2018; Li et al., 2019).

In conclusion, several toolboxes have already been tested on human footage of walking and running. Among the software packages in Table 3, *OpenPose* has been most extensively evaluated both in 2D and 3D gait estimation. In 2D video analysis, mean absolute errors of temporal parameters are smaller than differences arising from natural variations in the walking pattern making temporal changes detectable in healthy gait (Stenum et al., 2021). Step length estimation accuracy depends on the participant's position in the camera field of view, with central positions resulting in lower error rates. Unlike gait speed that reaches accuracy levels similar to the gold standard, errors in sagittal hip, knee, and ankle angles are in proximity of test-retest errors in the same plane. In an underwater running setup, the accuracy of predictions for 2D-joint marker positions extracted with *DeepLabCut* seems to match manual labels with a mean difference of fewer than three pixels (Cronin et al., 2019). Although not compared to a marker-based system, *DeepLabCut* seemed sensitive enough to differentiate between closely-spaced running cadences with a high test-retest reliability of the mean stride data. In 3D motion capture obtained with *OpenPose, DeepLabCut*, and *AlphaPose*, significant kinematic differences at hip and knee occurred in comparison to marker-based systems (Needham et al., 2021). Here, tracking accuracy of the ankle unexpectedly performed better than other joints, possibly owing to more precise manual annotation during training due to its apparent anatomical position. When compared to IMUs, *OpenPose* seems to exhibit tracking discrepancies in joint angles of up to 14 (Gu et al., 2018; D'Antonio et al., 2020, 2021). Despite these reports, Sato et al. (2019) employed a pipeline with *OpenPose* to analyze cadence in daily clinical movies recorded from the frontal angle in healthy controls (*n* = 117) and two PD patients with prominent FoG. The authors reported a discrimination performance for mild PD gait from controls of 0.75–0.96 (area under curve) and for comparing gait sequences before vs. after DBS treatment (*n* = 1) of 0.98. On the whole, as the demand for efficient and cost-effective technologies for gait analysis grows, deep learning architectures are still lacking in precision but continue to improve rapidly and are increasingly being implemented into clinical studies and home assessments (Xue et al., 2018; Viswakumar et al., 2019; Sibley et al., 2021).

### 4.3. Summary and Discussion

Non-wearable technologies are becoming an attractive tool for gait and balance analysis due to their advantages compared to wearables. Their availability, portability, easy setup, and complete non-intrusiveness shorten the preparation time significantly and may reduce the stress of the participant. These attributes yield comparatively low pricing, bringing non-wearable marker-less tools distinct advantages over customary gold standard technologies that are costly and difficult to deploy in environments of everyday activities. Conversely, vision-based motion tracking accuracy of non-wearable systems remains lower than that of marker-based systems. While discrepancies in temporal parameters stay at a small scale, spatial parameter differences including joint angles vary from system to system and are significantly influenced by the experimental environment. Indeed, the error rate of most systems depends on the recording conditions as well as movement complexity and speed, which limits data capture to greater constraints and reduces the number of feasible walking assessments. Moreover, depth camera technology remains sensitive to potential light interference from multiple sensors and operates only in certain volume ranges, reducing the amount of suitable settings (Colyer et al., 2018).

At present, the application of in-depth technology in neurological disorders to quantify gait and balance impairments is yet in exploration. Although the performance of the reviewed systems has been exploited in healthy gait, studies on validity and reliability in pathological gait patterns are still rare, especially for most recent developments (*Azure*). Existing studies showed that spatiotemporal and kinematic parameters from walking and standing can be extracted and used for differentiation between PD/MS individuals and neurologically intact controls (Behrens et al., 2014; Ťupa et al., 2015; Gholami et al., 2016; Eltoukhy et al., 2017; Sabo et al., 2020), as well as for falling, tremor, and freezing detection in PD (Bigy et al., 2015). Furthermore, combining the in-depth camera output with downstream ML methods seems promising for robust gait analysis in the clinical context (Ťupa et al., 2015; Bethoux et al., 2018; Elkurdi et al., 2018; Rocha et al., 2018). However, this comes at the loss of simplicity, and requires expert knowledge in the application.

Intensive research is currently carried out in the area of pose estimation with standard cameras. Yet, the available methods appear too complex for human gait analysis to be applied outside research environments at the moment. Nonetheless, the rapid evolution of these techniques can be predicted due to the high availability of video material and the already distributed toolboxes under creative commons licenses. Once intensively trained networks on large, standardized data sets are available, the application in clinical and home environments will be possible on a larger scale. In conclusion, for current and planned studies on movement disorders, the careful recording of video material, ideally from two or more perspectives, should be an integral part as this could allow a detailed motion analysis in the near future.

Altogether, marker-less vision-based motion tracking offers an exciting new opportunity for capturing gait-related data in the clinical context. Even though the technology is not yet mature, it shows distinct advantages over gold standard methods and might help unfold a new niche of easily accessible, repeated, longitudinal data collection not only in clinical but patients' home environments. The steady transition toward simpler recording technologies also fits impeccably the contact restrictions in the ongoing COVID-19 pandemic pushing the need for remote video measurements and analysis in telemedicine (Sibley et al., 2021).

## 5. Conclusion and Future Directions

This review examined established and emerging wearable and vision-based portable technologies for objective gait and balance analysis applicable for neurological disorders. The literature published on the topic is extensive reflecting the high demand for reliable, sensitive, easily accessible, easy to use, and mobile measurement systems. New developments aim to reduce monetary and personnel costs, improve accessibility, and allow short as well as long-term assessments in and outside the clinic. Meeting all these demands still poses a challenge, since the continuous detection and characterization of locomotion in various environments is a complex task. Nonetheless, a great number of gait and posture parameters can be captured with inertial sensors, instrumented insoles, smartphones, in-depth cameras, and also to some extent with standard camera technology. Due to the increased sensitivity of these objective parameters, early subtle gait dysfunction or disease progression become measurable (Horak et al., 2015). Therefore, instrumentalized gait and balance analysis will play a major role in prospective diagnosis, prevention, therapy, and monitoring of neurological disorders.

The decision on a suitable measurement and analysis tool for current studies and clinical examinations depends on balancing the requirements for validity, reliability, and usability. The first step is to define the key parameters that are to be measured with high accuracy and sensitivity with respect to the target group and their gait and movement characteristics. For example, step length was shown to be an important biomarker in PD and vision-based tracking methods might be more reliable than wearables in tracking this parameter (de-la Herran et al., 2014). Especially in joint angle tracking, the reviewed technologies still lack reliability compared to laboratory-based systems, which offer the greatest sensitivity and are reliable over a wide spectrum of measures. Secondly, the choice of a measurement instrument is heavily influenced by the given or desired measurement setup. The various technical solutions also offer different operating concepts and workflows. Parameters can be either extracted in real-time, thus being available for immediate biofeedback or adaptive therapies, or parameters are determined offline, often yielding a higher accuracy. The distinct usability aspects must be carefully weighed before deciding to integrate a system into clinical trials, workflows, or home applications.

Provided with the broad overview of literature in this review, we recommend a number of improvements for future research: (1) To overcome the existing inconsistencies in application, reporting, and interpretation of the extracted gait and balance measures, the utilized hardware and software, including the version number, should be reported. (2) When comparing the assessed parameters with values from the literature, one has to be very careful regarding the exact definition of the parameter calculation. At the moment, reported gait and balance parameters vary greatly between studies, making it difficult to compare treatment effects or to choose meaningful parameters for future investigations. Therefore, publications should provide the exact parameter definitions and methods in the supplements, when using self-implemented algorithms, or refer to applied definitions from literature (Benedetti et al., 2013; Siragy and Nantel, 2018). (3) For the same reasons, in any gait data analysis, gait velocity should be included in experiments as a final common expression of gait performance, plus a range of gait variables according to pre-defined criteria (Lord et al., 2013). (4) Due to rapid developments in pose estimation, the careful recording of video material, ideally from two or more perspectives, should be an integral part of any upcoming study as this material could allow a detailed motion analysis soon.

However, before the new systems are integrated into clinical routines, further research into the validity and reliability of each device is essential, preferably with comparative studies in large populations of neurologically intact controls and individuals with neurological disorders (Horak et al., 2015). This requirement contrasts with the advantage that wearable and marker-less vision-based systems are less expensive than gold-standard technologies: The more effort that has been put into the development and validation of a technology, the more expensive commercially available systems become. Despite the required improvements, we hold the opinion that portable systems for objective assessment of gait and balance characteristics are indispensable to support the neurological, face-to-face exam along with imaging and other biomarkers to facilitate individualized, adaptive treatments in the future. We see that there is a vicious circle to be escaped where as long as the technologies for simple and reliable gait analysis are not yet mature, the search for disease-specific biomarkers will be held up yielding skepticism toward the usefulness of these techniques. One future direction is the integration of several and novel sensor modalities (Buckley et al., 2019; Espay et al., 2019; Morita et al., 2020). Multiple sensors can provide redundant information and their fusion might reduce uncertainty, which can increase reliability in case of a sensor failure. The different modalities can provide objective, real-world data about the clinical phenotypes of individual patients over flexible amounts of time, possibly boosting our knowledge of locomotion and disease pathologies in the concept of deep phenotyping (Dorsey et al., 2020). The creation of normative databases (big data approaches) will yield an increased understanding of pathologies, enhancing the evaluation of therapies, and improve patient care (Buckley et al., 2019; Monje et al., 2019). In the long term, the emerging techniques for gait and balance tracking might be used for continuous monitoring and predicting disability such as fall risks in real-world environments (Weiss et al., 2015) and can be integrated into new, personalized therapeutic interventions. In the context of tele-consultations, tele-therapy and -rehabilitation, wearable and vision-based technologies can be utilized to report and monitor movement conditions and compliance with treatments. In the development of these telemedical applications, a strong focus should be on usability such that target user groups suffering from motor as well as mild cognitive impairments can use the technologies safely and reliably.

## Ethics Statement

Written informed consent was obtained from the individual(s) for the publication of any potentially identifiable images or data included in this article.

## Author Contributions

CS-H, MS, and MJ drafted the work (Introduction: CS-H, Measures of Gait and Postural Control and Wearable Technologies: CS-H and MJ, Non-wearable Technologies: Vision-Based Motion Analysis: MS and CS-H, Conclusion and Future Directions CS-H). All authors contributed to the conception, design of this work, and revised it critically for important intellectual content. All authors gave final approval of the version to be published and agree to be accountable for all aspects of the work.

## Funding

This work was funded by the German Federal Ministry of Education and Research (BMBF) within the project Mobil4Park—project ID FKZ16SV81 68—and the Deutsche Forschungsgemeinschaft (DFG, German Research Foundation)—project ID 424778381—TRR 295. NW is a Freigeist-Fellow supported by the Volkswagen Foundation, and a participant in the BIH Charité Clinician Scientist Program. NW and MS are supported by Hertie Academy of Clinical Neuroscience.

## Conflict of Interest

Author TS is co-founder of the SensorStim Neurotechnology GmbH, a company developing sensor-based stimulation technologies. The remaining authors declare that the research was conducted in the absence of any commercial or financial relationships that could be construed as a potential conflict of interest.

## Publisher's Note

All claims expressed in this article are solely those of the authors and do not necessarily represent those of their affiliated organizations, or those of the publisher, the editors and the reviewers. Any product that may be evaluated in this article, or claim that may be made by its manufacturer, is not guaranteed or endorsed by the publisher.

## Footnotes

^1^Unified Parkinson's Disease Rating Scale - Part III (UPDRS-III).

^2^Expanded Disability Status Scale (EDSS) 0–2.5 (Benedetti et al., 1999; Martin et al., 2006) and 2.5–6 (Remelius et al., 2012); Hauser Ambulation Index (HAI) 0–2 (group 1) and 3–4 (group 2) (Kelleher et al., 2010).

^3^https://developer.apple.com/documentation/coremotion/getting_raw_accelerometer_events [accessed July 6, 2021].
